# Comprehensive Analysis of MICALL2 Reveals Its Potential Roles in EGFR Stabilization and Ovarian Cancer Cell Invasion

**DOI:** 10.3390/ijms25010518

**Published:** 2023-12-30

**Authors:** Tianxiang Xia, Fengwen Ye, Weizhen Zhao, Pengxiang Min, Chenxiang Qi, Qianwen Wang, Mingyu Zhao, Yujie Zhang, Jun Du

**Affiliations:** Department of Physiology, Nanjing Medical University, Nanjing 211166, China; xiatianxiang21@163.com (T.X.); yefengwen1998@163.com (F.Y.); zhaowz_0809@163.com (W.Z.); minpx@njmu.edu.cn (P.M.); qcxdeyouxiang@yeah.net (C.Q.); sandyqianwenwang@163.com (Q.W.); zhaomy0330@163.com (M.Z.); zeater87@126.com (Y.Z.)

**Keywords:** MICALL2, ovarian cancer, invasion, EGFR degradation

## Abstract

Molecules interacting with CasL (MICALs) are critical mediators of cell motility that act by cytoskeleton rearrangement. However, the molecular mechanisms underlying the regulation of cancer cell invasion remain elusive. The aim of this study was to investigate the potential role of one member of MICALs, i.e., MICALL2, in the invasion and function of ovarian cancer cells. We showed by bioinformatics analysis that MICALL2 expression was significantly higher in tissues of advanced-stage ovarian cancer and associated with poor overall survival of patients. MICALL2 was strongly correlated with the infiltration of multiple types of immune cells and T-cell exhaustion markers. Moreover, enrichment analyses showed that MICALL2 was involved in the tumor-related matrix degradation pathway. Mechanistically, MMP9 was identified as the target gene of MICALL2 for the regulation of invadopodium formation and SKOV3, HO-8910PM cell invasion. In addition, EGFR–AKT–mTOR signaling was identified as the downstream pathway of MICALL2 in the regulation of MMP9 expression. Furthermore, MICALL2 silencing promoted EGFR degradation; however, this effect was abrogated by treatment with the autophagy inhibitors acadesine and chloroquine diphosphate. Silencing of MICALL2 resulted in a suppressive activity of Rac1 while suppressing Rac1 activation attenuated the pro-EGFR, pro-MMP9, and proinvasive effects induced by the overexpression of MICALL2. Collectively, our results indicated that MICALL2 participated in the process of immune infiltration and invasion by ovarian cancer cells. Moreover, MICALL2 prevented EGFR degradation in a Rac1-dependent manner, consequently leading to EGFR–AKT–mTOR–MMP9 signaling activation and invadopodia-mediated matrix degradation.

## 1. Introduction

Ovarian cancer is the most lethal gynecologic malignancy worldwide with nearly 314,000 new cases and more than 207,000 deaths recorded in 2020 [1]. It is an aggressive disease, and the invasion of ovarian cancer cells into surrounding tissue results in the development of widespread metastatic lesions throughout the peritoneal cavity. Ovarian cancer cell invasion contributes to abdominal discomfort and is associated with the pathogenesis of the abdominal mass and related organ damage. Despite great efforts in clinical and medical research, 65% of patients with ovarian cancer eventually succumb to this disease. Accordingly, knowledge of key proteins that influence ovarian cancer invasion can assist in elucidating the mechanisms underlying ovarian cancer metastasis and contribute to the identification of new targets for the treatment of this disease.

Cytoskeleton dynamics, regulated by various actin-binding proteins, play an important role in cell migration and invasion [2,3]. Therefore, the molecules interacting with the CasL (MICALs) family are attracting considerable research attention as potential contributors to cancer progression. In humans, the MICAL family of proteins consists of MICAL1, MICAL2, MICAL3, MICAL-like protein 1 (MICALL1), and MICALL2. MICAL1–3 contains a flavin adenine dinucleotide (FAD) domain and possesses flavoprotein oxidoreductase activity. However, MICALL1 and MICALL2 lack this region and do not produce reactive oxygen species [4]. It has been shown that MICALL2 interacts with the DENN-domain-containing 2B (DENND2B) at the cell periphery, thereby regulating the dynamic remodeling of the cell’s leading edge [5]. This process regulates the collective cell migration, allowing myriad cancer cells to behave as a single unit [6]. MICALL2 is highly expressed in several types of human epithelial cancers [7,8,9]. It is also positively correlated with the activation of heat shock protein 27/cytoskeleton (HSP27/cytoskeleton) and HSP27/beta-catenin pathways to accelerate gastric cancer cell migration [9]. Additionally, it was observed that the silencing of MICALL2 prevented the activation of canonical wnt/beta-catenin signaling and induced mesenchymal–epithelial transition (MET) in ovarian cancer cells [10]. However, to the best of our knowledge, the effects of MICALL2 on ovarian cancer cell invasion and the mechanisms responsible for this association remain unclear.

Matrix metalloproteinases (MMPs) play an important role in cancer cell invasion by mediating the degradation of the extracellular matrix (ECM). MMP9, a 92-kDa type IV collagenase, was highly expressed in patients with advanced ovarian cancer and correlated with poor prognosis [11]. The silencing of MICAL1 and MICAL2 reduced MMP9 expression in oral squamous cell carcinoma and breast cancer cells respectively [12,13]. In addition to proinflammatory factors, MMP9 expression is also regulated by several other growth factors, including epidermal growth factor/epidermal growth factor receptor (EGF/EGFR) activation [14,15]. A recent study revealed that MICALL2 contributes to the cell division cycle 42-dependent (Cdc42-dependent) EGFR stability and promotes gastric cancer cell migration [9]. Consequently, we hypothesized that MICALL2 may promote EGFR-signaling activation and MMP9 expression in ovarian cancer cells.

In the present study, we used bioinformatics analysis and immunohistochemical assays to investigate the expression of MICALL2 in ovarian cancer tissue samples. The objective was to determine its clinicopathological significance. Subsequently, we evaluated the role of MICALL2 in ovarian cancer carcinogenesis.

## 2. Results

### 2.1. MICALL2 Was Overexpressed in Human Ovarian Cancer Samples

**Figure 1:**
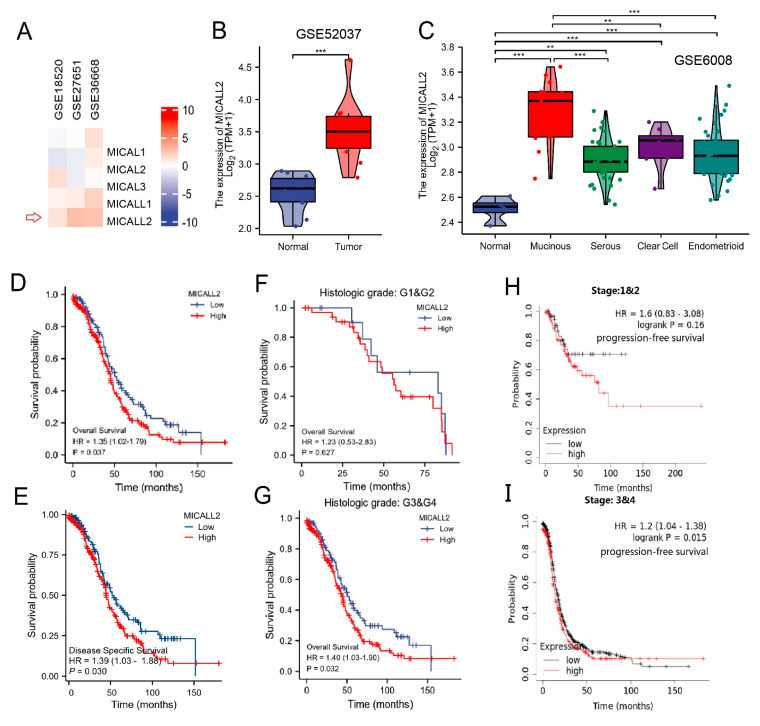
MICALL2 expression in ovarian cancer tissues. (**A**) Differences in the expression of MICALs in ovarian cancer, using data from databases. (**B**) Differences in MICALL2 expression between ovarian cancer tissue samples and normal tissue samples, using data from the GSE52037 dataset. (**C**) Differences in MICALL2 expression between subtypes of ovarian cancer, using data from the GSE6008 dataset. (**D**,**E**) Kaplan–Meier analysis of overall survival (OS) (**D**) and disease–specific survival (DSS) (**E**) for patients with low or high levels of MICALL2 expression. (**F**,**G**) Kaplan–Meier analysis of OS in the subgroup of patients with ovarian cancer. Grade 1 (G1) and G2 (**F**); G3 and G4 (**G**). (**H**,**I**) Kaplan–Meier analysis of progression–free survival (PFS) in the subgroup of patients with ovarian cancer. Tumor stage T1 and T2 (**H**); T3 and T4 (**I**). ** *p* < 0.01, *** *p* < 0.001.

To investigate whether MICALs were associated with the pathogenesis and progression of human ovarian cancer, we first assessed the mRNA levels of MICALs in several Gene Expression Omnibus datasets. We found that MICALL2 expression was significantly higher in tumor tissues versus normal tissues (Figure 1A). We further compared MICALL2 expression between ovarian cancer tissue and normal tissue using data from the GSE52037 dataset, obtaining similar results (Figure 1B). Analysis of the GSE6008 dataset showed that MICALL2 expression was significantly higher in mucinous ovarian, serous, clear cell, and endometrioid types of ovarian cancer than in normal ovarian mucosa (Figure 1C). A Kaplan–Meier survival curve analysis indicated that patients with high levels of MICALL2 expression were linked to markedly shorter overall survival and disease-specific survival versus those with low levels (Figure 1D,E). In addition, high MICALL2 expression was associated with poor overall survival of patients with grade 3–4 ovarian cancer (Figure 1F,G) and poor progression-free survival of patients with stage T3–T4 ovarian cancer (Figure 1H,I). Overall, these data indicated that MICALL2 expression is upregulated in ovarian cancer, and this increased expression may be associated with poor prognosis among patients with ovarian cancer.

### 2.2. Function Enrichment of MICALL2 in Ovarian Cancer

**Figure 2:**
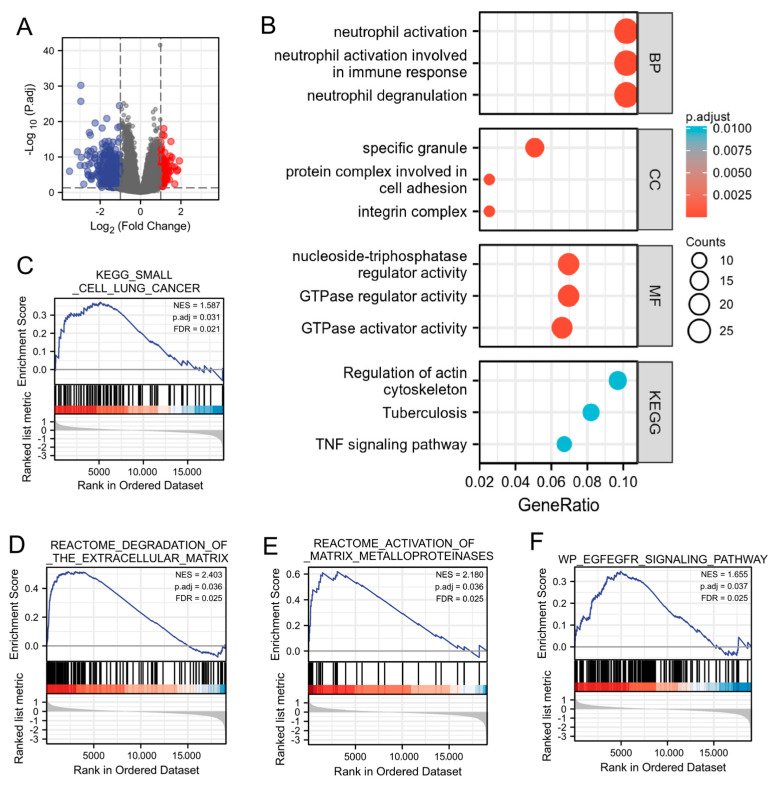
Function enrichment of MICALL2 in ovarian cancer by several analyses (GO, KEGG, and GSEA). (**A**) Volcano plot of differentially expressed genes between the MICALL2 high− and low− expression groups. (**B**) Enrichment plots from the GO and KEGG analyses. (**C**–**F**) Enrichment plots from the GSEA.

Subsequently, we investigated the possible cellular mechanism involved in the function of MICALL2 through KEGG and GSEA. Differentially expressed genes that correlated with MICALL2 were identified and used for gene ontology terms and KEGG analyses. As shown in Figure 2A, by analyzing the dataset from TCGA, we identified 528 differentially expressed genes (|log fold-change| > 1, adjusted *p*-value < 0.05) between the MICALL2 high- and low-expression groups, including 93 upregulated and 435 downregulated genes. The following biological processes were significantly affected: neutrophil activation; neutrophil activation involved in immune response; and neutrophil degranulation. The cellular component terms were mainly enriched in a specific granule, protein complex involved in cell adhesion, and integrin complex. The molecular function terms were mainly enriched in nucleoside-triphosphatase regulator activity, GTPase regulator activity, and GTPase activator activity. The KEGG terms were mainly involved in the regulation of the actin cytoskeleton, tuberculosis, and the tumor necrosis factor (TNF) signaling pathway (Figure 2B). The GSEA results showed that the coexpressed genes were involved in MMPs, ECM degradation, and the EGFR signaling pathway (Figure 2C–F). Therefore, we further investigated immune infiltration and the matrix-degradation pathway to better understand the function of MICALL2 in ovarian cancer.

### 2.3. Association between MICALL2 and Immune Infiltration

**Figure 3:**
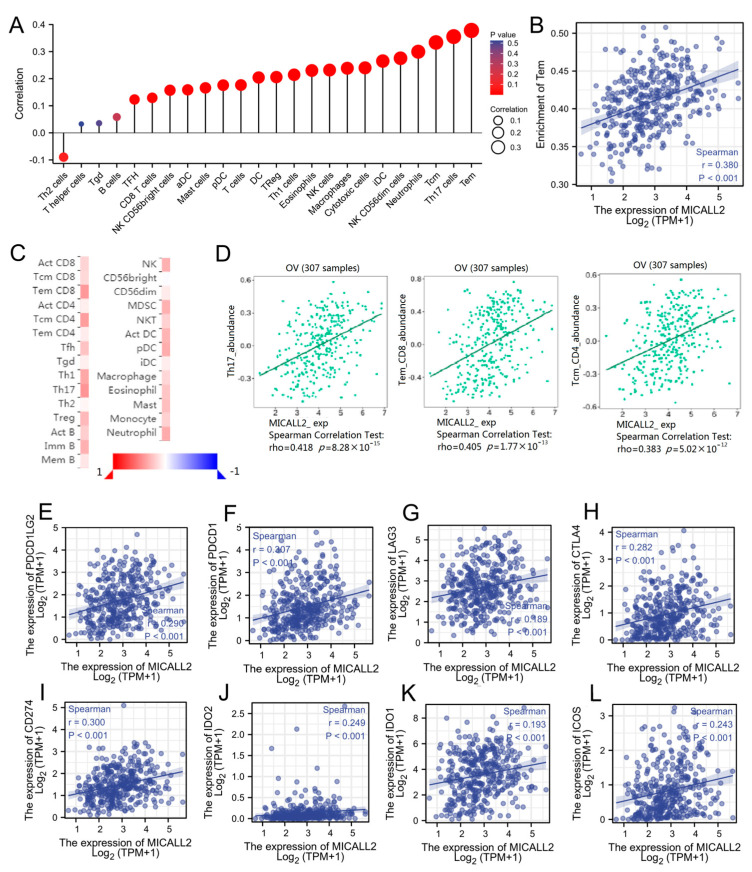
Correlation between MICALL2 and immune filtration in ovarian cancer. (**A**) Correlations between MICALL2 expression and immune infiltration by ssGSEA. n = 381. (**B**) Correlation analysis of the enrichment of Tem cells and the expression of MICALL2. n = 381. (**C**,**D**) Correlations between MICALL2 and immune infiltration, using data from the TISIDB database. n = 307. (**E**–**L**) Correlations between MICALL2 and T−cell exhaustion markers in ovarian cancer. n = 303.

Initially, we explored the association between MICALL2 expression and immune-cell infiltration in ovarian cancer through single-sample GSEA. MICALL2 was positively correlated with the infiltration levels of most types of immune cells, particularly effector memory T (Tem) cells. However, it was negatively associated with the infiltration level of T helper 2 (Th2) cells (Figure 3A,B). We also used TISIDB to explore the correlations between MICAL1 expression and the infiltration of various types of immune cells, yielding consistent results (Figure 3C,D). Furthermore, the results obtained from TIMER implied that programmed cell death 1 (PDCD1), lymphocyte activating 3 (LAG3), cytotoxic T-lymphocyte-associated protein 4 (CTLA4), programmed cell death 1 ligand 2 (PDCD1LG2), and other T-cell exhausting markers, are strongly correlated with MICALL2 expression (Figure 3E–L). Therefore, the results demonstrated that MICALL2 was closely correlated with the regulation of T-cell exhaustion in ovarian cancer.

### 2.4. Effect of MICALL2 on Matrix Degradation in Ovarian Cancer Cells

**Figure 4:**
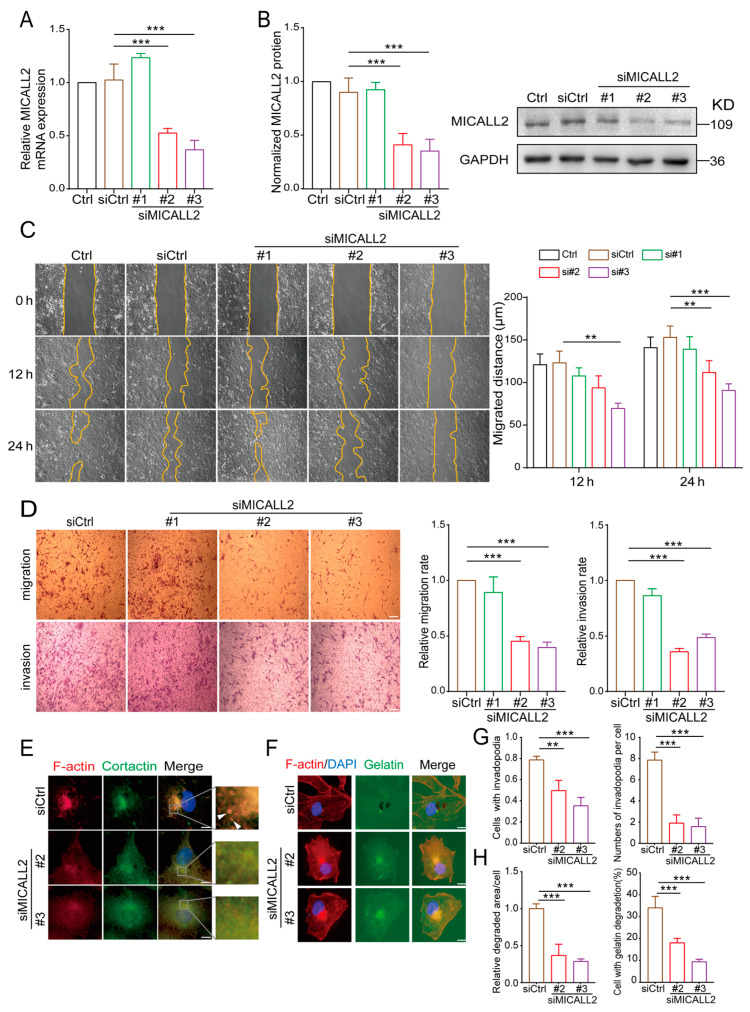
Effects of MICALL2 on the invasive ability of human ovarian cancer cells. (**A**,**B**) SKOV3 cells were transfected with control siRNA or siRNA-targeting MICALL2 (siMICALL2). Total mRNA (**A**) and protein (**B**) extracts from these cells were analyzed to determine the expression of MICALL2. Western blotting bands corresponding to MICALL2 were quantified, and the obtained values were normalized against the levels of GAPDH (n = 3 per group). (**C**) Wound-healing assays were carried out; SKOV3 cells were transfected with siRNA and siMICALL2, and the cell-migration rates were determined (n = 8 per group). (**D**) Representative images of transwell assays in SKOV3 cells transfected with control siRNA or siMICALL2, and quantifications of the cell-migration and invasion rates were determined (n = 5 per group). Scale: 50 μm. (**E**) After transfection with siRNA and siMICALL2, SKOV3 cells were stained with phalloidin (red), DAPI (blue), and cortactin (green). Scale: 2 μm. (**F**) Different groups of SKOV3 cells were plated on FITC-gelatin (green) and stained with phalloidin (red) and DAPI (blue). Scale: 2 μm. (**G**) Quantification of the percentage of cells with invadopodia and the number of invadopodia per cell. (**H**) Quantification of the percentage of cells degraded in gelatin and the relative degradation area of each cell. ** *p* < 0.01, *** *p* < 0.001.

To confirm the role of MICALL2 in the regulation of cell migration and invasion, we performed MICALL2 loss-of-function assays in ovarian cancer cells. First, we silenced MICALL2 expression in SKOV3 cells using siMICALL2. The knockdown efficiency was determined by quantitative PCR and Western blotting. As shown in Figure 4A,B, transfection with siMICALL2 #2 and #3 led to a significant reduction in MICALL2 expression in SKOV3 cells compared to control cells. Next, the effects of MICALL2 depletion on ovarian cancer cell migration and invasion were evaluated by wound healing and transwell assays. The results showed that silencing of MICALL2 effectively impaired the migratory and invasive potential of SKOV3 cells (Figure 4C,D). To determine the cellular functions of MICALL2 in promoting tumor invasion, we tested whether the expression of MICALL2 was associated with an increased ability to degrade ECM. MICALL2 silencing significantly prevented the formation of invadopodia in SKOV3 and HO-8910PM cells (Figure 4E,G; Figure S1A,B). Furthermore, SKOV3 cells were plated onto an FITC-conjugated gelatin matrix to assess their abilities for matrix degradation. We found that suppression of MICALL2 resulted in a marked reduction in matrix degradation (Figure 4F,H).

### 2.5. Effect of MICALL2 on MMP9 Expression

**Figure 5:**
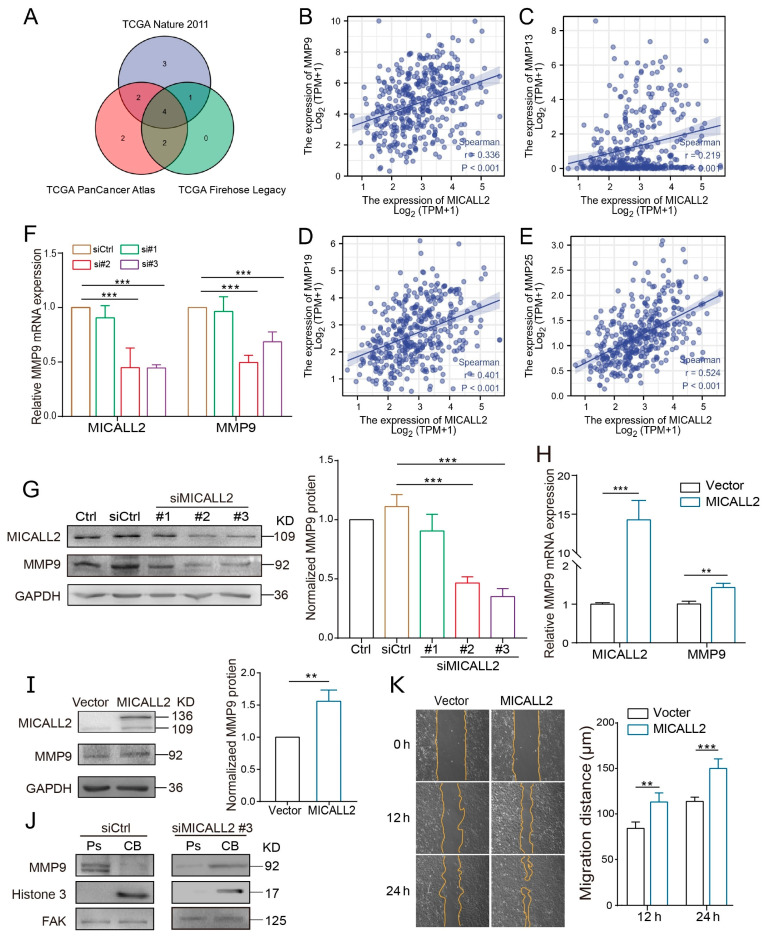
Effect of MICALL2 on MMP9 expression. (**A**) Screening of MICALL2-related MMP family genes, using data from the TCGA database (https://www.cbioportal.org/, accessed on 7 May 2022). (**B**–**E**) Correlation analysis between MMP9, MMP13, MMP19, MMP25, and MICALL2. (**F**) The mRNA expression of MMP9 in SKOV3 cells transfected with siRNA and siMICALL2 was detected by qPCR. (**G**) The mRNA expression of MMP9 in SKOV3 cells transfected with empty plasmid and MICALL2-overexpression plasmid was detected by qPCR. (**H**) Western blotting was used to detect the total protein expression of MMP9 in SKOV3 cells transfected with siRNA and siMICALL2. (**I**) The total protein expression of MMP9 in SKOV3 cells transfected with empty plasmid and MICALL2-overexpression plasmid was detected by Western blotting. (**J**) MMP9 protein expression in the cell bodies (CB) and pseudopodia (Ps) of SKOV3 cells transfected with siRNA and siMICALL2 was measured using Western blotting. (**K**) Wound-healing assays were carried out; SKOV3 cells were transfected with empty plasmid and MICALL2-overexpression plasmid, and quantification of the cell-migration rate was carried out (n = 8 per group). ** *p* < 0.01, *** *p* < 0.001.

It is well established that MMPs play important roles in maintaining the ability for matrix degradation. We sought to explore whether MICALL2 affects the expression of MMPs in ovarian cancer cells. For this purpose, we initially examined the impact of MICALL2 on the mRNA levels of MMPs using data from The Cancer Genome Atlas database. The Venn diagram revealed a positive correlation of the genes MMP9, MMP13, MMP19, and MMP25 with MICALL2 in several groups (Figure 5A–E). Both invadopodium formation and ECM degradation are induced by MMP9. Therefore, we next tested whether MICALL2 modulates the expression of MMP9 in ovarian cancer cells SKOV3. We noticed a marked reduction in the mRNA and protein levels of MMP9 in SKOV3 cells upon MICALL2 silencing (Figure 5F,G). As expected, overexpression of MICALL2 in SKOV3 cells led to increased MMP9 mRNA and protein expression relative to that measured in control cells (Figure 5H,I). According to these findings, MICALL2-mediated modulation of MMP9 expression may be dependent on transcription. Furthermore, the silencing of MICALL2 impaired the localization of MMP9 in invadopodia and the migration rate in SKOV3 cells (Figure 5J,K). These data indicated that the impairment of invasion induced by MICALL2 silencing was at least partly mediated by decreased invadopodium formation and function, which depends on MMP9 expression in SKOV3 cells.

### 2.6. Expression of MICALL2 and MMP9 in Ovarian Cancer Tissues

**Figure 6:**
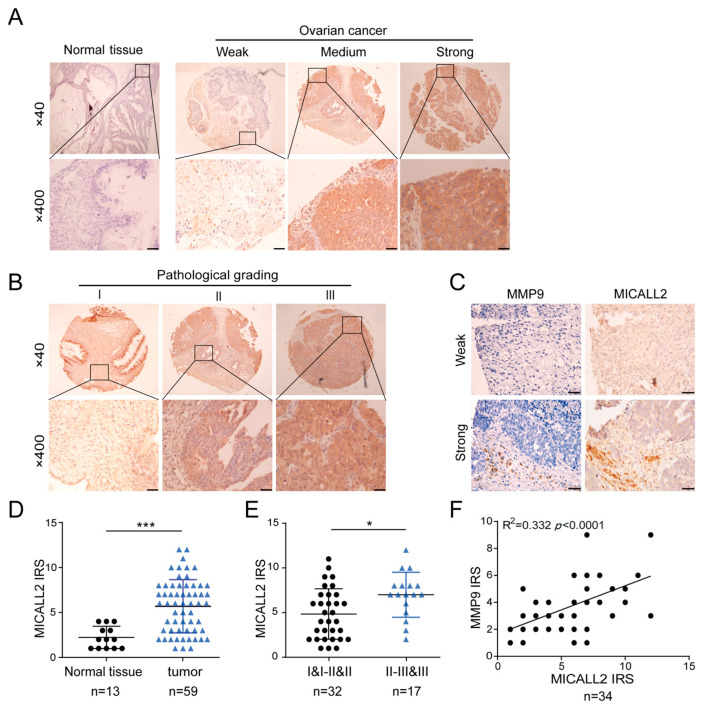
Analysis of MICALL2 and MMP9 expression in ovarian cancer tissues. (**A**) Representative images of MICALL2 staining in ovarian cancer and paracancerous tissues. MICALL2-positive staining is brown, and the nucleus is stained with hematoxylin. Scale: 20 μm. (**B**) Representative images of MICALL2 staining in ovarian cancer tissues of different grades. Scale: 20 μm. (**C**) Using continuous slices of the same sample, representative ovarian cancer and adjacent tissues stained for MICALL2 and MMP9 are displayed. (**D**,**E**) Analysis of MICALL2 staining in ovarian cancer tissues. Scale: 50 μm. (**F**) Analysis of MICALL2 and MMP9 staining correlation in ovarian cancer tissue through IRS. * *p* < 0.05, *** *p* < 0.001.

To investigate whether our in vitro experimental results are consistent with the pathogenesis of ovarian cancer, we examined the expression of MICALL2 and MMP9 in ovarian cancer and adjacent tissues using a microarray (30 paired cases). Immunohistochemistry results indicated that MICALL2 was highly expressed in tumor tissues compared with matched paracancerous tissues (Figure 6A,D). Histological examination also revealed that MICALL2 staining in the higher-grading group was significantly greater than that noted in the lower-grading group (Figure 6B,E). Furthermore, immunostaining of MICALL2 and MMP9 in the samples revealed a positive correlation in terms of expression (R2 = 0.332, *p* < 0.0001) (Figure 6C,F).

### 2.7. Effect of MICALL2 on EGFR Autophagy

**Figure 7:**
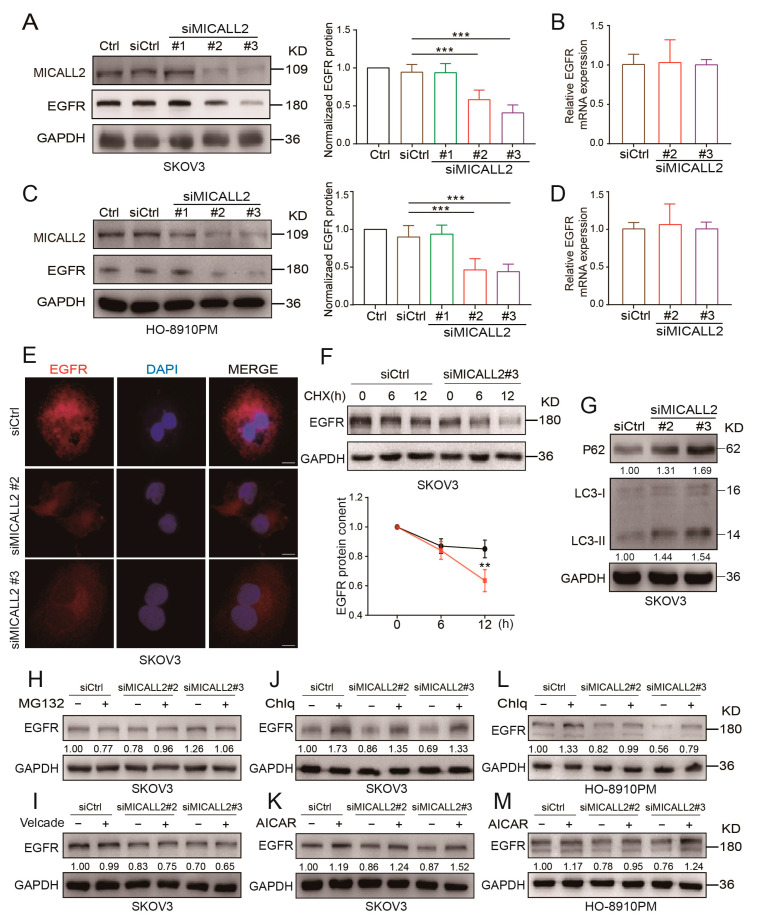
Effect of MICALL2 on EGFR expression. (**A**–**D**) Detection of EGFR mRNA and protein expression in SKOV3 (**A**,**B**) and HO-8910PM (**C**,**D**) cells transfected with siRNA and siMICALL2. (**E**) Representative immunofluorescence images of different groups of SKOV3 cells stained for EGFR. Scale: 10 μm. (**F**) After blocking protein synthesis with cycloheximide (CHX; 10 μg/mL) for the indicated times, the cells were lysed, and the levels of EGFR were determined. (**G**) P62 and LC3 expression in SKOV3 cells transfected with siRNA and siMICALL2 was detected by Western blotting. (**H**–**M**) After transfection with siRNA and siMICALL2, SKOV3 and HO-8910PM cells were treated with MG132 (20 µM), bortezomib (Velcade; 10 µM), Chlq (10 µM), and AICAR (0.2 mM) for 12 h. Subsequently, the levels of EGFR were detected. ** *p* < 0.01, *** *p*< 0.001.

We also sought to uncover the potential mechanism underlying the induction of MMP9 expression through MICALL2 silencing. Thus, we tested the EGFR levels in ovarian cancer cells according to the results obtained from the bioinformatics analysis. As shown in Figure 7A,C,E, MICALL2 depletion in both SKOV3 and HO-8910PM cells significantly inhibited EGFR protein expression, as well as its distribution in the cytoplasm and membrane. However, it did not affect the relative levels of EGFR mRNA expression in SKOV3 and HO-8910PM cells (Figure 7B,D). As shown in Figure 7F, MICALL2 depletion in SKOV3 cells significantly promoted EGFR degradation following the addition of CHX (a protein synthesis blocker) to the culture medium. These findings suggested that MICALL2-mediated modulation of EGFR expression is dependent on EGFR degradation.

The Western blotting results verified the increased expression of LC3-II and P62 protein in SKOV3 MICALL2-silenced cells versus control cells (Figure 7G), suggesting that MICALL2 silencing induced notable disturbances in autophagy. We also noticed that chloroquine diphosphate (an autophagy inhibitor) could block MICALL2 knockdown-induced invadopodia extensions and cell invasion (Figure S2A,B). We sought to further uncover the mechanisms involved in MICALL2-mediated degradation of EGFR. Thus, we treated cells with two kinds of inhibitors linked to established degradation pathways, namely MG-132 and bortezomib (Velcade) (proteasome inhibitors) and chloroquine and acadesine (autophagy inhibitors). The results showed that only the autophagy inhibitors could reverse the EGFR degradation induced by the knockdown of MICALL2 (Figure 7H–K). Similar results were observed with HO-8910PM cells (Figure 7L,M). These results suggested that MICALL2 inhibits EGFR degradation possibly by preventing its entry into the autophagy pathway.

### 2.8. MICALL2 Regulated EGFR Stability via Rac1 Activation

**Figure 8:**
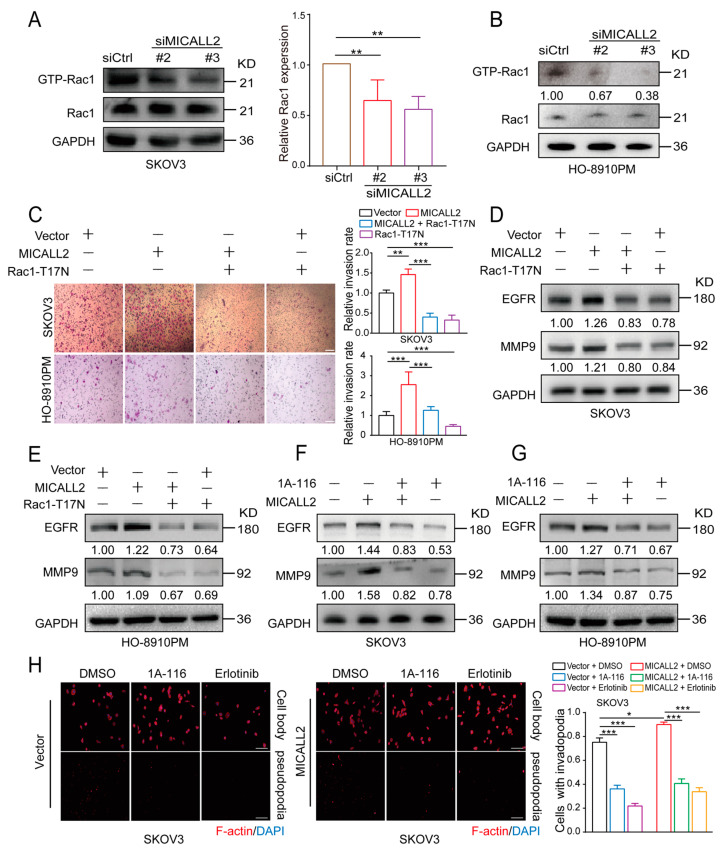
MICALL2 regulated EGFR stability through Rac1. (**A**,**B**) SKOV3 (**A**) and HO-8910PM cells (**B**) were transfected with siMICALL2. Thereafter, the activity of Rac1 was measured by a pull-down assay. (**C**–**E**) MICALL2-overexpressing SKOV3 and HO-8910PM cells were transfected with the Rac1-T17N plasmid. Next, the cell-migration rate (**C**) and the protein levels of EGFR and MMP9 (**D**,**E**) were determined. (**F**,**G**) MICALL2-overexpressing SKOV3 (**F**) and HO-8910PM cells (**G**) were pretreated with 1A-116 (Rac1 inhibitor). Thereafter, the protein levels of EGFR and MMP9 were determined. (**H**) MICALL2-overexpressing SKOV3 cells were pretreated with 1A-116 and erlotinib (EGFR inhibitor). Subsequently, the number of invadopodia/cell was counted. Scale: 50 μm. * *p* < 0.05, ** *p* < 0.01, *** *p* < 0.001.

It has been reported that Rac1 plays an important role in the internalization and degradation of receptors. To further investigate the mechanism through which the silencing of MICALL2 reduces EGFR degradation, we examined Rac1 activity by pulldown assays in both SKOV3 and HO-8910PM cells. We found that Rac1 activity was significantly reduced by MICALL2 knockdown (Figure 8A,B). Furthermore, marked increases in the cell-invasion rate, MMP9 and EGFR expression levels, and invadopodia formation were found in MICALL2-overexpressing ovarian cells. These effects were reversed by ectopic expression of Rac1-T17N (a constitutively inactive mutant of Rac1) (Figure 8C–E), as well as pretreatment with 1A-116 (Rac1 inhibitor) or erlotinib (EGFR inhibitor) (Figure 8F–H). These findings demonstrated that MICALL2 stabilized the EGFR levels and invasive ability of ovarian cancer cells in a Rac1-dependent manner.

### 2.9. Effect of MICALL2 on EGFR–AKT–mTOR Signaling

**Figure 9:**
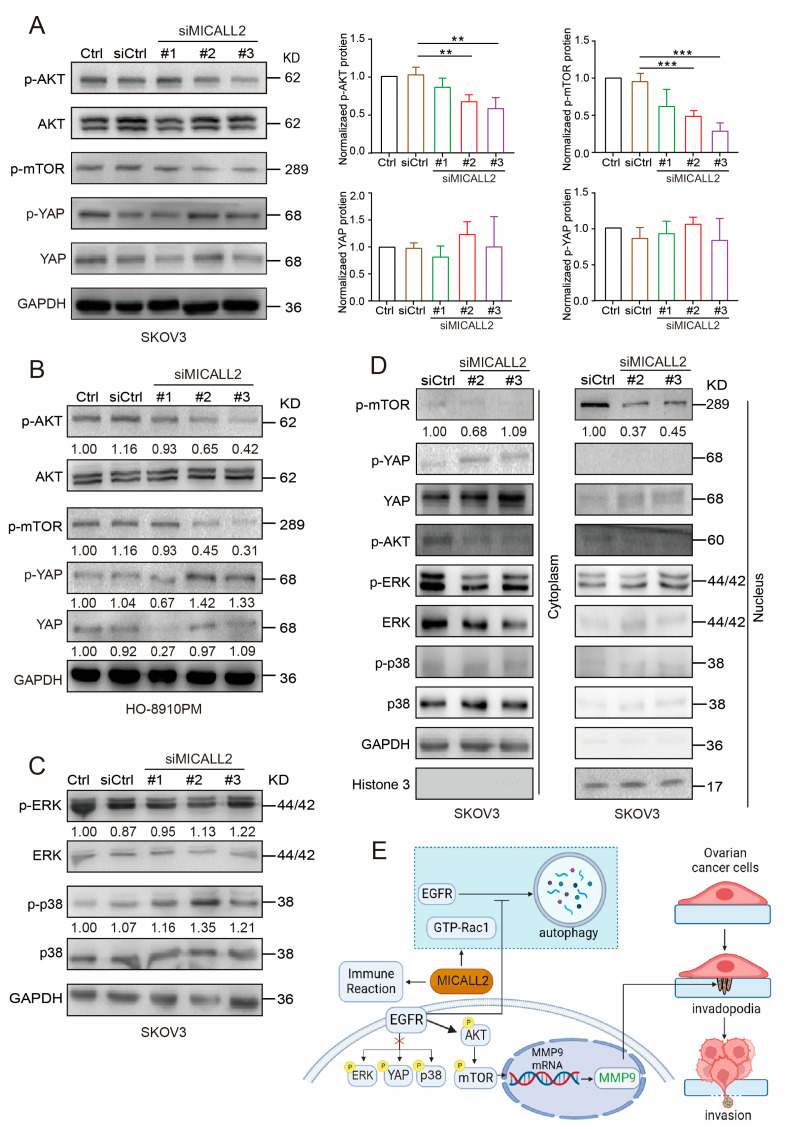
Effect of MICALL2 on the AKT–mTOR signaling pathway. (**A**,**B**) SKOV3 (**A**) and HO-8910PM (**B**) cells were transfected with control siRNA or siMICALL2. Subsequently, the levels of p-AKT, p-mTOR, and p-YAP were detected by Western blotting. n = 3 for (**A**). (**C**) SKOV3 cells were transfected with control siRNA or siMICALL2. Thereafter, the levels of p-ERK and p-p38 were detected. (**D**) The levels of p-mTOR, p-YAP, YAP, p-AKT, p-ERK, and p-p38 were measured in cytoplasmic and nuclear extracts obtained from SKOV3 cells transfected with siMICALL2. GAPDH and histone H3 served as the cytoplasmic and nuclear control, respectively. (**E**) A diagram (created with BioRender.com) was shown. MICALL2 markedly inhibits EGFR degradation by the autophagy pathway in a Rac1-dependent manner; in turn, this effect promotes EGFR accumulation and activation of the EGFR–AKT–mTOR pathway, while increasing the nuclear translocation of mTOR. These processes result in the upregulation of MMP9 expression, which stimulates invadopodia formation and ovarian cancer cell invasion. MICALL2 stimulates MMP9 expression, which may be independent of ERK, YAP, and p38 activation/inactivation. MICALL2 also influences immune reaction and closely correlates with T-cell exhaustion in ovarian cancer. ** *p* < 0.01, *** *p* < 0.001.

To further confirm the relationship between MICALL2 and EGFR signaling, we investigated whether its downstream effectors were altered following MICALL2 depletion. Western blotting analysis showed that p-EGFR and AKT/mTOR phosphorylation levels were markedly reduced in both SKOV3 and HO-8910PM cells upon MICALL2 depletion (Figure 9A,B, Figure S3A,B). MICALL2 depletion increased the amount of p-p38; it did not exert a significant effect on p-ERK. YAP phosphorylation was increased in MICALL2-depleted HO-8910PM cells, but not in MICALL2-depleted SKOV3 cells (Figure 9A–C). As shown in Figure 9D, the depletion of MICALL2 led to a reduction in mTOR levels in nuclear fractions. Collectively, these findings indicated that MICALL2 selectively modulates the activation of the EGFR–AKT–mTOR pathway. It is established that nuclear translocation of mTOR can activate MMP9 transcription [16]. Hence, these findings implied that MICALL2 markedly inhibits EGFR degradation; in turn, this effect promotes activation of the EGFR–AKT–mTOR pathway, while increasing the nuclear translocation of mTOR. These processes result in the upregulation of MMP9 expression, which stimulates invadopodia formation and ovarian cancer cell invasion. Overall, our clinical and in vitro data supported that MICALL2 may participate in the process of immune reaction and promote ovarian cancer cell invasion via the EGFR pathway (Figure 9E).

## 3. Discussion

In a previous study, we suggested that MICALL2 is highly expressed in colon adenocarcinoma and could be a promising biomarker for determining the poor prognosis of patients with this disease [7]. Similarly, the findings of the present study demonstrated that upregulation of MICALL2 was associated with malignant phenotypes in patients with ovarian cancer, probably through matrix degradation and immune infiltration pathways. Moreover, we identified a novel link between MICALL2 and MMP9 in the regulation of invadopodia formation and the invasion of ovarian cancer cells. Specifically, MICALL2 maintains the stability of EGFR protein in a Rac1-dependent manner, thereby enhancing the activation of the EGFR–AKT–mTOR signaling pathway and MMP9 expression.

It is well established that metastatic cancer cells invade surrounding tissues through the formation of invadopodia. This process leads to the degradation of the ECM, thereby enabling the passage of cancer cells through it. MMP9 is required for invadopodia maturation and distant metastasis [17,18]. The present bioinformatics analysis showed that MMP9 was a target of MICALL2. Moreover, the depletion of MICALL2 inhibited the mRNA and protein expression of MMP9. This effect was accompanied by a reduction in the invasive ability of cancer cells. Notably, overexpression of MICALL2 reversed these effects. Additionally, MICALL2 silencing significantly prevented the formation of invadopodia and reduced the levels of MMP9 in invadopodia. These findings indicate that MICALL2 promotes cancer cell invasion by stimulating MMP9 expression in a transcription-dependent manner. Endogenous MMP2 also plays a role in promoting ovarian cancer cell invasion [19,20]. However, we observed that the mRNA levels of MMP2 were not altered after the knockdown of MICALL2 (data not shown).

Our bioinformatics analysis also showed that EGFR signaling was a target of MICALL2. EGFR may not be a prognostic biomarker for patients with ovarian cancer [21]. Nevertheless, targeting EGFR has been useful in the treatment of ovarian cancer [22,23,24,25]. EGFR localization in invadopodia has also been observed in breast cancer cells [26]. It was previously reported that ubiquitination of EGFR reduced its levels in multiple types of cells [27,28,29]. However, in this study, we found that MICALL2 attenuated EGFR degradation by preventing its entry into the autophagy pathway rather than the ubiquitin–proteasome system. Based on these findings, we hypothesize that the functions of MICALL2 in promoting ovarian cancer cell invasion might be mediated by the stabilization of EGFR expression and, consequently, the activation of EGFR downstream signal pathways. Thereafter, a number of regulated proteins in the downstream signaling pathway of EGFR were identified by examining the mechanism through which MICALL2 induced MMP9 transcription in ovarian cancer cells.

The major MMP9-related molecular pathways downstream of EGFR include AKT–mTOR, ERK, p38, STAT3, YAP, etc. [13,30,31,32]. Although MICALL2 stabilized EGFR expression, it only stabilized the activation of its downstream AKT–mTOR signaling pathway in ovarian cancer cells. MICALL2 negatively regulated the activation and nuclear localization of p38; however, it did not exert a significant effect on p-ERK. In addition, the results regarding YAP phosphorylation status after the knockdown of MICALL2 in the two ovarian cancer cells were inconsistent. We abandoned the idea of exploring the YAP pathway further. Genetic heterogeneity between the different cell lines might explain the inconsistent results. A possible explanation for this observation is that the inhibitory effects of some EGFR downstream effectors caused by the silencing of MICALL2 may be counteracted by compensatory effects in ovarian cancer cells. For instance, adequate YAP signaling pathway inactivation and ERK, p38 signaling pathway activation are necessary to maintain cancer cell survival. It is well established that mTOR phosphorylation greatly contributes to the expression of MMP9 and cell invasion [33,34]. AKT–mTOR signaling upregulates MMP9 expression by promoting H3K27Ac and H3K56A on the MMP9 promoter region [16]. Knockdown of MICALL2 inhibited AKT and mTOR phosphorylation; it is suggested that knockdown of MICALL2 inhibits EGFR expression and reduces the endogenous activation of the AKT–mTOR signaling pathway, thereby downregulating MMP9 mRNA and protein expression.

This study also explored the mechanism through which MICALL2 regulates the stabilization of EGFR. Rac1 is a member of the Rho GTPase family, which also includes RhoA and Cdc42. It has been shown that Rac1 is involved in an array of biological functions, including cell motility and tumor metastasis [35,36], as well as endocytic and exocytic transport [37]. In addition, Rac1 acts as a negative modulator of autophagy. For example, active Rac1 competes with LC3, thus preventing its appropriate recruitment to autophagosomes [38,39]. Active Rac1 also functions as a negative modulator of autophagy by targeting TFEB (a master regulator of autophagy) [39]. In ovarian cancer cells, silencing of MICALL2 greatly suppressed Rac1 activation, whereas MICALL2 overexpression exerted the opposite effect. These results indicated that MICALL2 also stabilizes Rac1 activation in ovarian cancer cells. We also analyzed the role of Rac1 in the maintenance of EGFR stability. We observed that both dominant negative mutants of Rac1 (T17N) and Rac1 activation inhibitor 1A-116 could block the high levels of EGFR and MMP9 induced by the overexpression of MICAL2. Silencing of MICALL2 significantly increased EGFR autophagy. Thus, it is likely that MICALL2 prevents EGFR autophagy in a Rac1-dependent manner. The mechanisms by which MICALL2 regulates Rac1 activation remain to be elucidated.

MICALL2 is a critical factor in promoting immune infiltration and T-cell exhaustion in the renal tumor microenvironment [40]. Consistently, our observations clearly demonstrated that MICALL2 also exerts distinct effects on immune responses. It was found that MICALL2-overexpressing ovarian cancer cells attract multiple immune cells into the tumor. Moreover, MICALL2 significantly enhanced the expression of T-cell exhaustion markers, such as PDCD1, LAG3, CTLA4, etc. Tem cells express integrins and chemokine receptors, which are necessary for their translocation to inflamed tissues [41]. Lieber et al. also showed that CD8+ Tem cells in ascites differed vastly among patients. In addition, the number of cells in this fluid was positively correlated with the survival of patients with ovarian cancer. However, following the migration of Tem cells into the tumor microenvironment, their activation is suppressed by mediators (phospholipase C gamma 1 [PLCγ1] and signal transducer and activator of transcription 5B [STAT5B]) [42]. Further investigation is warranted to verify these results and to accurately understand the relationship between MICALL2 and immune infiltration within the microenvironment of ovarian cancer.

In conclusion, the findings of this study propose a new mechanism through which MICALL2 participates in the process of immune infiltration and matrix degradation by ovarian cancer cells. MICALL2 was positively correlated with immune-cell infiltration and T-cell exhaustion markers. Furthermore, MICALL2 strengthened the stability of EGFR, consequently leading to the activation of EGFR–AKT–mTOR–MMP9 signaling and invadopodia-mediated matrix degradation. We further showed that this effect of MICALL2 on EGFR stabilization may depend on Rac1 activation, which prevents autophagy of EGFR. To the best of our knowledge, this was the first study that investigated the mechanism of MICALL2 in mediating MMP9 expression in cancer cells. Further advances in our understanding of the function of MICALL2 may have important implications for the treatment of ovarian cancer.

## 4. Materials and Methods

### 4.1. Ethics Statement

All immunohistochemical assays involving human tumor specimens were conducted according to the institutional guidelines of Jiangsu Province.

### 4.2. Cell Culture

Human ovarian cancer cell lines SKOV3 and HO-8910PM were purchased from the Cell Biology Institute of the Chinese Academy of Science (Shanghai, China). All cells were maintained in Dulbecco’s modified Eagle’s medium (Hyclone; Thermo Fisher Scientific, Waltham, MA, USA) containing 10% fetal bovine serum (Gibco, Carlsbad, CA, USA) and incubated at 37 °C in a humidified incubator with 5% CO_2_.

### 4.3. Plasmids and siRNAs

The empty vector control pcDNA-3.1-GFP-C and full-length human MICALL2 cDNA were purchased from YouBio (Changsha, China). The siRNAs used in this study were synthesized and purified by GenePharma (Shanghai, China). The sequences of the siRNAs targeting MICALL2 were siRNA targeting MICALL2 (siMICALL2) #1, 5′-GGUUCCCA CAAAGAGUAUATT-3′; siMICALL2 #2, 5′-CUCGACGUUUGUGACAACUTT-3′; and siMICALL2 #3, 5′-CCAAGUUCCGCUUGUCCAATT-3′. Transfection (plasmids or siRNA) was performed using a Lipofectamine 2000 (Thermo Fisher Scientific) according to the instructions provided by the manufacturer. Cells were harvested 48 h after transfection and analyzed by Western blotting or other assays, as specified below for each experiment.

The transfected cells were treated with cycloheximide (CHX) (HY-12320; MedChemExpress [MCE], Shanghai, China), MG-132 (HY-13259; MCE), bortezomib (Velcade; HY-10227; MCE), acadesine (HY-13417; MCE), chloroquine diphosphate (HY-17589A; MCE), 1A-116 (HY-104064; MCE), and erlotinib (SC0168; Beyotime, Shanghai, China) at the indicated time points.

### 4.4. Cell-Migration and Invasion Assay

For the wound-healing assay, cells were seeded in six-well plates until they reached confluence. A wound was performed in the cell monolayer using a 10-µL pipette tip. After rinsing with phosphate-buffered saline, the cells were allowed to migrate for the indicated time. Images of wound areas were captured using an inverted phase-contrast microscope (Carl Zeiss Meditec, Jena, Thuringen, Germany). The objective lens was 40X and the eye lens was 10X when we observed cell migration.

The 24-well cell-culture chambers with 8 μm pores were used to determine the migratory and invasive capabilities of the cells. Chambers precoated with Matrigel (356234; Corning Inc., Corning, NY, USA) were used for the evaluation of cell invasion. A total of 1 × 10^6^ cells were seeded in the upper chamber containing serum-free medium, while the lower chamber contained medium supplemented with 10% fetal bovine serum. After 24 h of incubation, nonmigrated cells on the upper side of the membrane were removed, and the remaining cells in the chambers were fixed in 4% paraformaldehyde and stained with 0.1% crystal violet. The average number of migrating or invading cells was counted in five randomly selected fields under the microscope.

### 4.5. Western Blotting

Western blotting analysis was conducted as previously described [9]. Bodies and pseudopodia proteins were obtained as previously described [43]. Cytoplasmic and nuclear protein fractions were obtained using a nuclear protein extraction kit (P0028, Beyotime). Membranes were cut horizontally according to the visible protein marker size. Primary antibodies against MICALL2 (24408-1-AP), EGFR (66455-1-Ig), MMP9 (27306-1-AP), phosphorylated-mechanistic target of rapamycin (p-mTOR; 67778-1-Ig), and Rac family small GTPase 1 (Rac1; 24072-1-AP) were purchased from Proteintech (Wuhan, China). The antibody against glyceraldehyde-3-phosphate dehydrogenase (GAPDH; BS72410) p-EGFR (AF5797) was obtained from Bioworld (Nanjing, China), while those against Yes1-associated transcriptional regulator (YAP; 4912), p-YAP (13008), protein kinase B (AKT; 4691), p-AKT (4060), p38 (9212), p-p38 (4511), ERK (4696), p-ERK (4370), and histone H3 (4499) were purchased from Cell Signaling Technology (Danvers, MA, USA).

Horseradish peroxidase-conjugated normal rabbit IgG (ZF0101, ZFanti, Nanjing, China) was used as a secondary antibody. The bands were visualized using an enhanced chemiluminescence reagent (FuDeBio, Hangzhou, China) and analyzed using Quantity One software (Bio-Rad, Hercules, CA, USA).

### 4.6. Real-Time Quantitative Polymerase Chain Reaction (PCR)

Real-time quantitative PCR was performed as previously described [8]. The sequences of the primers used in this experiment were GAPDH, 5′-TCGGATCAACGGATTTGGT-3′ (sense) and 5′-TTCCCGTTCTCAGCCTTGAC-3′ (antisense); MICALL2, 5′-TGTGGTCCAGAGGAGGAATGA-3 (sense) and 5′-CAGCTCCGGTGGTAAAGCC-3′ (antisense); MMP9, 5′-GGTGATTGACGACGCCTTTG-3′ (sense) and 5′-AAACCGAGTTGGAACCACGA-3′ (antisense); and EGFR, 5′-AGGCACGAGTAACAAGCTCAC-3′ (sense) and 5′-ATGAGGACATAACCAGCCACC-3′ (antisense).

### 4.7. Immunohistochemistry

Ovarian cancer tissue microarrays were purchased from Outdo Biotech (Shanghai, China). A total of 50 paraffin-embedded surgically resected cancerous tissues and the corresponding adjacent normal tissues were collected from patients with ovarian cancer from Nanjing Maternity and Child Health Care Hospital (Nanjing, China). Immunohistochemical staining was performed as previously described [44]. The primary antibodies used were MICALL2 (24408-1-AP; Proteintech) and MMP9 (27306-1-AP; Proteintech). Briefly, after dewaxing, hydration, heat-induced antigen retrieval, and blockage of endogenous peroxidase activity, the sections were incubated with primary antibodies overnight. After washing thrice with phosphate-buffered saline, the sections were incubated with the appropriate secondary antibody for 1 h. Thereafter, the sections were counterstained with hematoxylin. Images were captured with an Olympus BX51 microscope (Olympus, Tokyo, Japan). MICALL2 and MMP9 immunoreactivity were semiquantified using the immunoreactive score (IRS) [45].

### 4.8. Invadopodium Formation Assays

As previously reported [46], invadopodium formation was determined by immunofluorescent assays using rabbit anticortactin (AF2134; Beyotime), fluorescein isothiocyanate-conjugated (FITC-conjugated) goat antirabbit IgG (33106ES60; Yeasen, Shanghai, China) and Alexa Fluor 568-conjugated phalloidin (40734ES75; Yeasen). Colocalization of F-actin with cortactin was used to determine invadopodium formation.

For the monitoring of invadopodium function, cells were cultured for 4–8 h on coverslips preloaded with a mixture of FITC–gelatin (G13186; Thermo Fisher Scientific) and unlabeled gelatin and stained with Alexa Fluor 568-conjugated phalloidin and 4′,6-diamidino-2-phenylindole. Invadopodium-associated gelatin degradation was defined as the colocalization of F-actin puncta with gelatin degradation. To quantify the gelatin degradation activity of invadopodia, the degradation areas in the images were analyzed.

### 4.9. Protein Stability Assay

After incubation with CHX for 0, 6, and 12 h, the cells were collected and lysed. The lysates were separated by sodium dodecyl sulfate–polyacrylamide gel electrophoresis and analyzed through Western blotting to determine the protein abundance at each time point.

### 4.10. Pulldown Assay

Rho GTPase pulldown assays were performed as previously described [9]. Active Rac1 was pulled down using p21-activated kinase-Cdc42-Rac interactive binding (PAK-CRIB) beads. Briefly, cells were washed and lysed with lysis buffer. After centrifugation, the supernatants were mixed with beads precoupled with PAK-CRIB and incubated at 4 °C for 30 min. Thereafter, the beads were washed, and the proteins bound on the beads were separated by sodium dodecyl sulfate–polyacrylamide gel electrophoresis. The quantity of GTP-Rac1 was determined by Western blotting analysis using a mouse antibody against human Rac1. Total Rac1 was also determined by Western blotting.

### 4.11. Clinicopathological Analysis of MICALL2

Databases (The Cancer Genome Atlas and Gene Expression Omnibus), (https://tcga-data.nci.nih.gov/tcga/, accessed on 7 May 2022), (https://www.ncbinlm.nih.gov/geo/, accessed on 7 May 2022), (https://www.cbioportal.org/, accessed on 7 May 2022), the GSE52037, GSE6008, GSE18520, GSE27651, and GSE36668 datasets, and the Kaplan–Meier plotter (https://kmplot.com/, accessed on 7 May 2022) were used to analyze the association between mRNA expression of MICALL2 and the clinical parameters. Moreover, the prognostic value of MICALL2 mRNA expression in ovarian cancer tissues was also determined [47,48]. Serous ovarian carcinoma is the most common type of ovarian cancer. Then, we focused on this subtype. Patients with serous ovarian cancer were divided into MICALL2 high- and low-expression groups based on the median mRNA levels of MICALL2. The enriched pathways were detected using Gene Ontology (http://geneontology.org/, accessed on 7 May 2022) [49,50], the Kyoto Encyclopedia of Genes and Genomes (KEGG) (https://www.genome.jp/kegg, accessed on 29 May 2022) [51,52,53], and gene set enrichment analysis (GSEA) (https://www.gsea-msigdb.org/gsea, accessed on 29 May 2022) [54]. The predefined gene set for GSEA was from the MSigDB database (https://www.gsea-msigdb.org/gsea/msigdb/index.jsp, accessed on 29 May 2022).

Immune infiltration of ovarian cancer was identified using single-sample GSEA (ssGSEA) [54,55]. The infiltration levels of different immune-cell types were quantified from gene-expression profiles. In addition, a Spearman correlation was used to investigate the association of immune cells with MICALL2 expression. The Tumor Immune Estimation Resource (TIMER) (https://cistrome.shinyapps.io/timer/, accessed on 29 May 2022) was also used to evaluate immune infiltration [56]. TISIDB (http://cis.hku.hk/TISIDB/index.php, accessed on 29 May 2022), an integrated repository portal for tumor–immune system interactions, was used to investigate correlations between MICALL2 and immunoinhibitors [57].

### 4.12. Statistical Analysis

Data extracted from databases were analyzed via the R3.6.3 software [58]. Other data were analyzed using SPSS version 19.0 (IBM Corp., Armonk, NY, USA) and are reported as mean ± standard error of the mean. Student’s *t*-test was used for comparisons between the two groups. One-way analysis of variance was utilized for comparisons among three or more groups. A *p*-value < 0.05 denoted a statistically significant difference.

## Data Availability

The data that support the findings of this study are available from the corresponding author upon reasonable request.

## Supplementary Materials

The following supporting information can be downloaded at: https://www.mdpi.com/article/10.3390/ijms25010518/s1.
