# Supplementary material for: Comprehensive Analysis of MICALL2 Reveals Its Potential Roles in EGFR Stabilization and Ovarian Cancer Cell Invasion
Source: Int J Mol Sci. 2023 Dec 30;25(1):518. doi: 10.3390/ijms25010518 (PMC10778810; doi:10.3390/ijms25010518)
Supplement: Supplementary file 1 [file ijms-25-00518-s001.zip › ijms-2770773-supplementary.pdf]

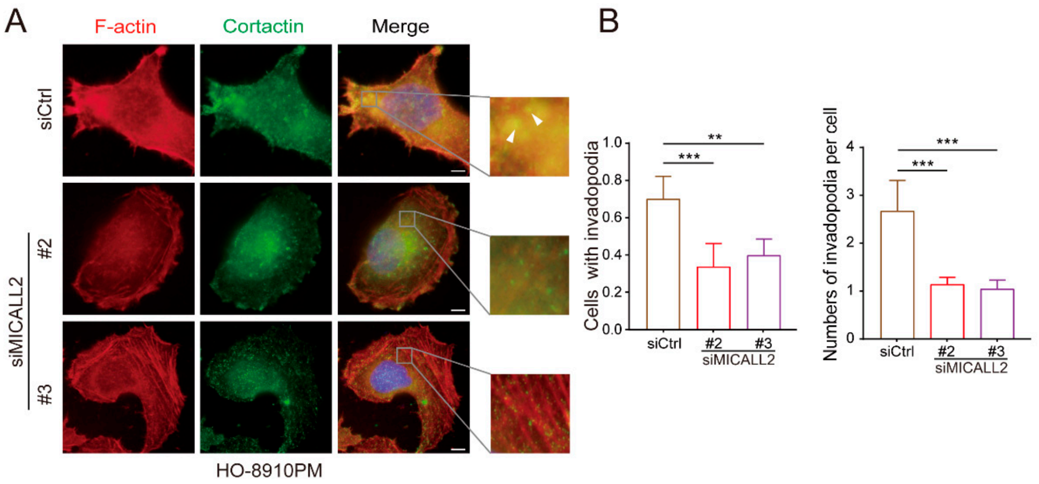

Figure S1 Effect of siMICALL2 on the invadopodia formation of HO-8910PM cells. (A) After transfection with siRNA and siMICALL2, HO-8910PM cells were stained with phalloidin (red), DAPI (blue), and cortactin (green). (B) Quantification of the percentage of cells with invadopodia and the number of invadopodia per cell. Scale: 2 μm. ***P*<0.01, ****P*<0.001.

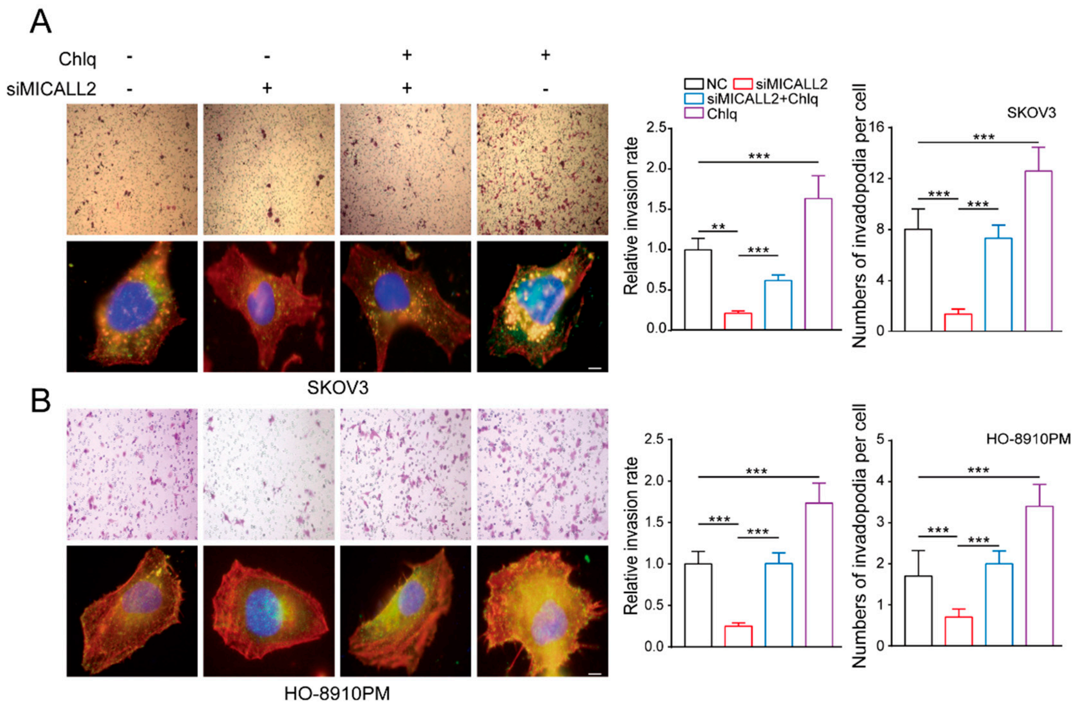

Figure S2 Effect of autophagy on the invasive ability of ovarian cancer cells. After the silencing of MICALL2, SKOV3 (A) and HO-8910PM cells (B) were pre-treated with Chlq (autophagy inhibitor). Subsequently, the invasive ability of cells and the number of invadopodia extensions in cells were determined. Scale: 2 μm. ****P*<0.001.

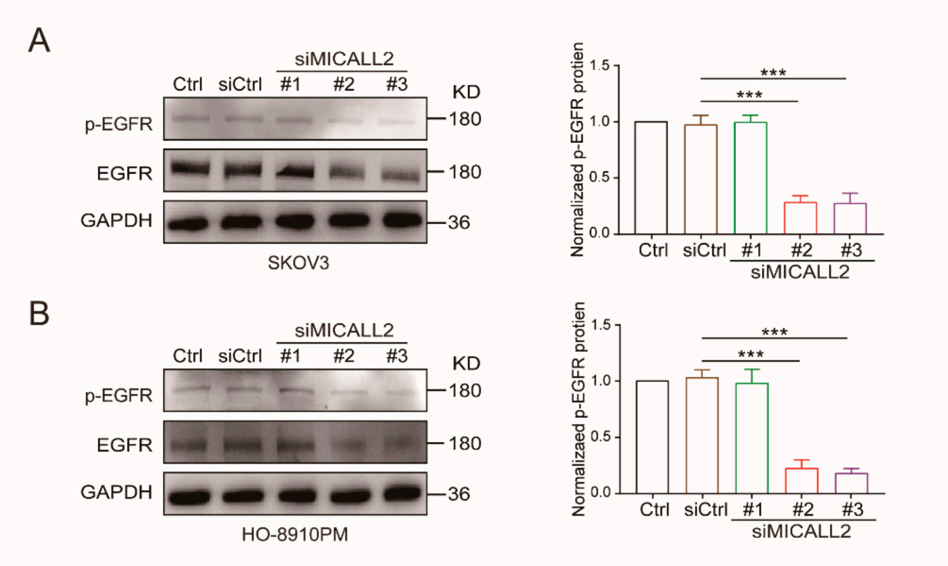

Figure S3 Effect of siMICALL2 on p-EGFR expression. (A, B) SKOV3 (A) and HO-8910PM (B) cells were transfected with control siRNA or siMICALL2. Subsequently, the levels of p-EGFR were detected by western blotting. ****P*<0.001.
